# Modified Guide-Assisted Percutaneous Trigger Finger Release Using a Custom-Made Kirschner Wire (K-wire): Long-Term Outcomes of a Retrospective Case Series From Gaza City

**DOI:** 10.7759/cureus.115574

**Published:** 2026-09-01

**Authors:** Sharif J Salha, Abdalla Y Dalwol, Nour A Dalwol

**Affiliations:** 1 Public Health, Al-Quds University, Jerusalem, PSE; 2 Orthopedics and Hand Surgery, Al-Shifa Medical Complex, Gaza City, PSE; 3 Medicine, Islamic University of Gaza, Gaza City, PSE

**Keywords:** a1 pulley, gaza, k-wire guide, minimally invasive surgery, percutaneous release, resource-limited setting, stenosing tenosynovitis, trigger finger

## Abstract

Background

Trigger finger (stenosing flexor tenosynovitis) is a common cause of hand pain and disability. Percutaneous release is a minimally invasive alternative to open surgery, but few guide-assisted, low-cost techniques have been described for resource-limited settings. This study evaluated the long-term outcomes, recurrence rate, safety, and patient satisfaction of a modified percutaneous trigger finger release using a custom-made Kirschner wire (K-wire) guide.

Methods

In this retrospective observational case series, 13 patients (26 trigger digits) treated between May 2016 and May 2026 at the Orthopedic Outpatient Clinic, Al-Shifa Medical Complex, Gaza City, Palestine, were reviewed. All procedures used a 16-18-gauge needle guided by a custom-made K-wire guide under local anesthesia in an outpatient setting. Outcomes assessed included immediate release, Quinnell grade, range of motion, Visual Analog Scale (VAS) pain score, recurrence, complications, and patient satisfaction.

Results

The mean age of patients was 50.5 ± 9.5 years (range 34-65 years); seven patients (53.8%) were male. Immediate release and complete resolution of triggering were achieved in all 26 digits (100%). At final follow-up (range: 3 months to 10 years), all digits demonstrated full range of motion, Quinnell grade 0, and a VAS score of 0. No recurrence, repeat procedure, or conversion to open surgery occurred. Overall patient satisfaction was 100% (11/13 very satisfied, 2/13 satisfied).

Conclusions

This modified percutaneous release technique using a custom-made K-wire guide demonstrated favorable long-term outcomes, with no recurrence and high patient satisfaction. It may represent a practical, minimally invasive, and resource-efficient outpatient technique for managing trigger finger, particularly in resource-limited settings.

## Introduction

Trigger finger, also known as stenosing flexor tenosynovitis, is one of the most common hand disorders encountered in orthopedic practice. The condition results from thickening of the flexor tendon sheath and narrowing at the level of the A1 pulley, leading to painful clicking, locking, or triggering during finger movement. It affects approximately 2-3% of the general population and is more common among middle-aged individuals, women, diabetic patients, and individuals exposed to repetitive hand activity [1]. Management ranges from conservative measures, including splinting, physiotherapy, nonsteroidal anti-inflammatory drugs, and corticosteroid injection, to surgical intervention in refractory cases. Open surgical release has historically been considered the definitive standard of care because of its high success rate; however, it may be associated with surgical scarring, postoperative pain, wound complications, prolonged recovery, and increased healthcare costs [2]. Percutaneous trigger finger release has gained increasing popularity as a minimally invasive outpatient alternative. Multiple studies have shown that percutaneous release allows faster functional recovery, earlier return to work, reduced postoperative pain, and shorter operative time, with clinical outcomes broadly comparable to open release [1,3]. Chanthanapodi and Aodsup reported a success rate of 97.6% with no recurrence or nerve injury on long-term follow-up [3], and Panghate et al. demonstrated significant improvement in Quinnell grading [4] and pain scores using a hypodermic-needle technique with no major complications [5]. Earlier systematic reviews, including that by Wang et al., reported similar findings [6]. A 2024 meta-analysis by Casey et al., comprising eight randomized controlled trials and 548 patients, found no significant differences between open and percutaneous techniques in treatment failure, revision rate, complications, or postoperative pain [7]. More recent work has focused on technical refinements and image guidance to improve procedural safety and precision. A 2025 randomized controlled trial by Öner et al. compared ultrasound-guided percutaneous release with open release in 146 patients; outcomes were broadly comparable at 12 months, but four revisions occurred in the ultrasound-guided group, related to incomplete release, recurrent tenosynovitis, flexor tendon rupture, and neurovascular injury [8]. This finding is notable because it demonstrates that even image-guided percutaneous release is not free of procedure-related risk. Moungondo et al. described a retrograde percutaneous technique using dedicated sono-instrumentation, emphasizing technical pearls to reduce incomplete release [9]. Ultrasound guidance, however, is frequently unavailable in resource-constrained healthcare environments. In settings such as the Gaza Strip, where access to operating theatres, imaging equipment, and surgical resources is frequently limited, a central open question is whether a simple, low-cost mechanical guide can provide sufficient procedural control over needle trajectory in the absence of ultrasound. Although percutaneous trigger finger release has been widely investigated, there is limited published evidence describing guide-assisted techniques designed to improve needle control using inexpensive and readily available materials [4,10-12]. This study reports the feasibility and observed clinical outcomes of a modified guide-assisted percutaneous release technique using a custom-made 1.8-mm Kirschner wire (K-wire) guide in patients treated at Al-Shifa Medical Complex in Gaza City, Palestine, over a 10-year period, and is intended to motivate future prospective, controlled comparison against free-hand percutaneous release.

## Materials and methods

Study design and setting

This was a retrospective observational case series conducted at the Orthopedic Outpatient Clinic, Al-Shifa Medical Complex, Gaza City, Palestine, and reported with attention to STROBE principles for observational studies where feasible given the constraints of the operating environment.

Study population

Patients were eligible for inclusion if they had clinically diagnosed symptomatic trigger finger that was refractory to conservative treatment, or if they elected surgical release after clinical evaluation and discussion of available management options. All patients treated with the modified percutaneous release technique between May 19, 2016, and May 19, 2026, were considered eligible. Thirteen patients, comprising 26 affected fingers and thumbs, met inclusion criteria and were included in the final analysis; because inclusion required refractoriness to conservative measures or elective surgical release, all treated digits were, by design, more advanced (Quinnell grade III-IV) at presentation, and no grade I-II digits were included in this cohort. Diagnosis was based on clinical history and examination findings of painful triggering, locking, or catching of the affected digit during active flexion and extension, with a palpable nodule over the A1 pulley. Patient selection and treatment flow are summarized in Figure 1.

**Figure 1:**
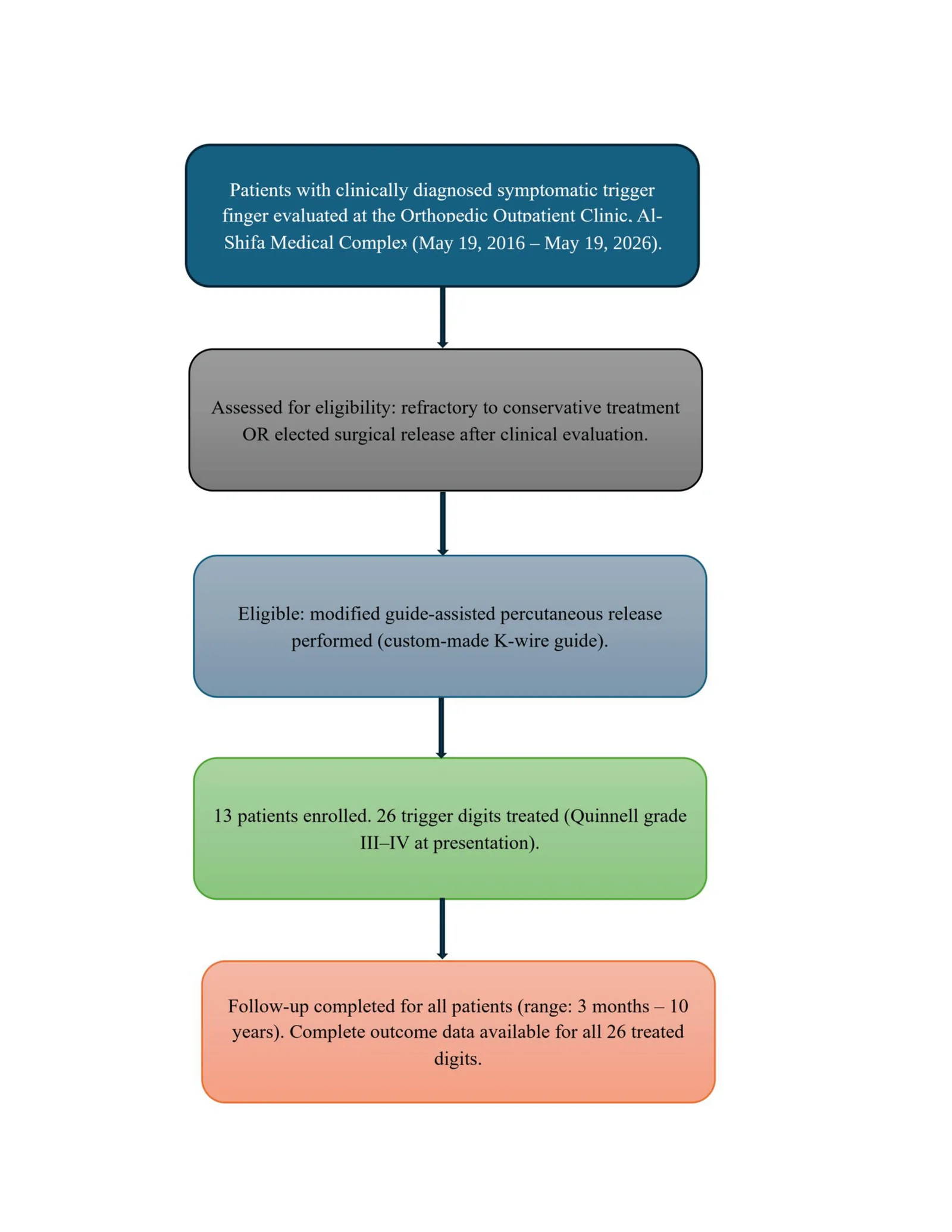
Patient selection and treatment flow Patient selection and treatment flow. Patients with clinically diagnosed symptomatic trigger finger evaluated at the Orthopedic Outpatient Clinic, Al-Shifa Medical Complex, between May 19, 2016, and May 19, 2026, were assessed for eligibility based on refractoriness to conservative treatment or elective surgical release. Thirteen patients (26 trigger digits) met inclusion criteria and underwent custom-made K-wire guide-assisted percutaneous release. All patients completed follow-up ranging from 3 months to 10 years, with complete outcome data available for all 26 treated digits. The figure was created using Microsoft Word (Microsoft Corp., Redmond, Washington, USA). K-wire: Kirschner wire

Technique history and data collection timing

The technique was first developed and used clinically by the senior author (A.Y.D.) beginning in May 2016 and was applied in its present standardized form throughout the study period. Preoperative Visual Analog Scale (VAS) scores [13] and Quinnell grades [4] were recorded contemporaneously in the clinical record at the time of treatment and at routine follow-up clinic visits for all 13 patients in this series. No preoperative or immediate postoperative outcome data were reconstructed retrospectively from memory or incomplete records; this case series reflects a retrospective analysis of prospectively documented clinical data. Four patients underwent the procedure before it was formally evaluated as a case series (see "Ethical Considerations"); their contemporaneously recorded clinical data were included using the same variables and definitions as the remainder of the cohort.

Demographic and clinical data were extracted from clinic records, including age, sex, occupation, healthcare-worker status, dominant hand, affected digit(s), relevant comorbidities (diabetes mellitus, rheumatoid arthritis, thyroid disease, hypertension, asthma), smoking status, symptom duration, preoperative and postoperative Quinnell grade, active range of motion, VAS pain score, complications, recurrence, and patient satisfaction.

Disease severity was graded using the Quinnell classification, a five-point clinical grading system for assessing trigger finger severity based on the degree of triggering observed on active finger movement [4]. The grades are defined as follows: Grade 0, normal active movement of the digit with no triggering; Grade I, uneven or irregular active movement of the digit; Grade II, triggering that the patient can actively correct; Grade III, triggering that requires passive correction by the examiner of the other hand; and Grade IV, a fixed, irreducible flexion deformity of the digit.

Surgical technique

All procedures were performed on an outpatient basis under local anesthesia using a single, disposable 16-gauge needle in conjunction with a custom-made K-wire guide designed to standardize needle entry point, trajectory, and depth during A1 pulley release. The guide is fashioned from a sterile 1.8-mm K-wire (Brasseler USA, Savannah, Georgia, USA), a smooth stainless-steel pin conventionally used for hand fracture fixation, bent to a fixed geometry approximately 1.8 cm in length that defines the entry point, angle, and depth of needle insertion. The guide was manufactured locally, is reused across cases following standard sterilization between uses, and has an approximate unit cost of $2 (USD).

The technique followed a standardized sequence: (1) The affected hand was prepared and draped in a sterile fashion, and the A1 pulley was localized by palpation at the level of the distal palmar crease (or the metacarpophalangeal flexion crease for the thumb). (2) A local anesthetic was infiltrated subcutaneously over the A1 pulley, avoiding direct infiltration into the tendon sheath. (3) The custom-made K-wire guide was positioned against the skin to define a fixed entry point, angle, and depth of needle insertion, constraining the needle trajectory to the level of the A1 pulley, kept parallel to the flexor tendon and away from the adjacent neurovascular bundles. (4) The 16-gauge needle was advanced along the guide using controlled longitudinal sweeping and levering movements to divide the A1 pulley, while the patient performed active finger flexion and extension to confirm progressive release. (5) Immediate procedural success was defined prospectively as complete disappearance of triggering during active flexion and extension immediately after release, confirmed clinically by the surgeon, together with a palpable give at the level of the pulley and full, smooth active range of motion. (6) A simple dressing was applied, and patients were discharged the same day with instructions for early active mobilization.

Procedure duration ranged from approximately 3 to 7 minutes per digit. The technique still requires sterile preparation, standard infection-control measures, trained personnel, appropriate sharps disposal, and sterilization of the reusable guide between uses, even though it does not require an operating theater.

A schematic of the guide-assisted needle trajectory relative to the A1 pulley and flexor tendon is shown in Figure 2. Two supplementary videos demonstrating the procedure, performed by the senior author (A.Y.D.), are provided in the Appendices.

**Figure 2:**
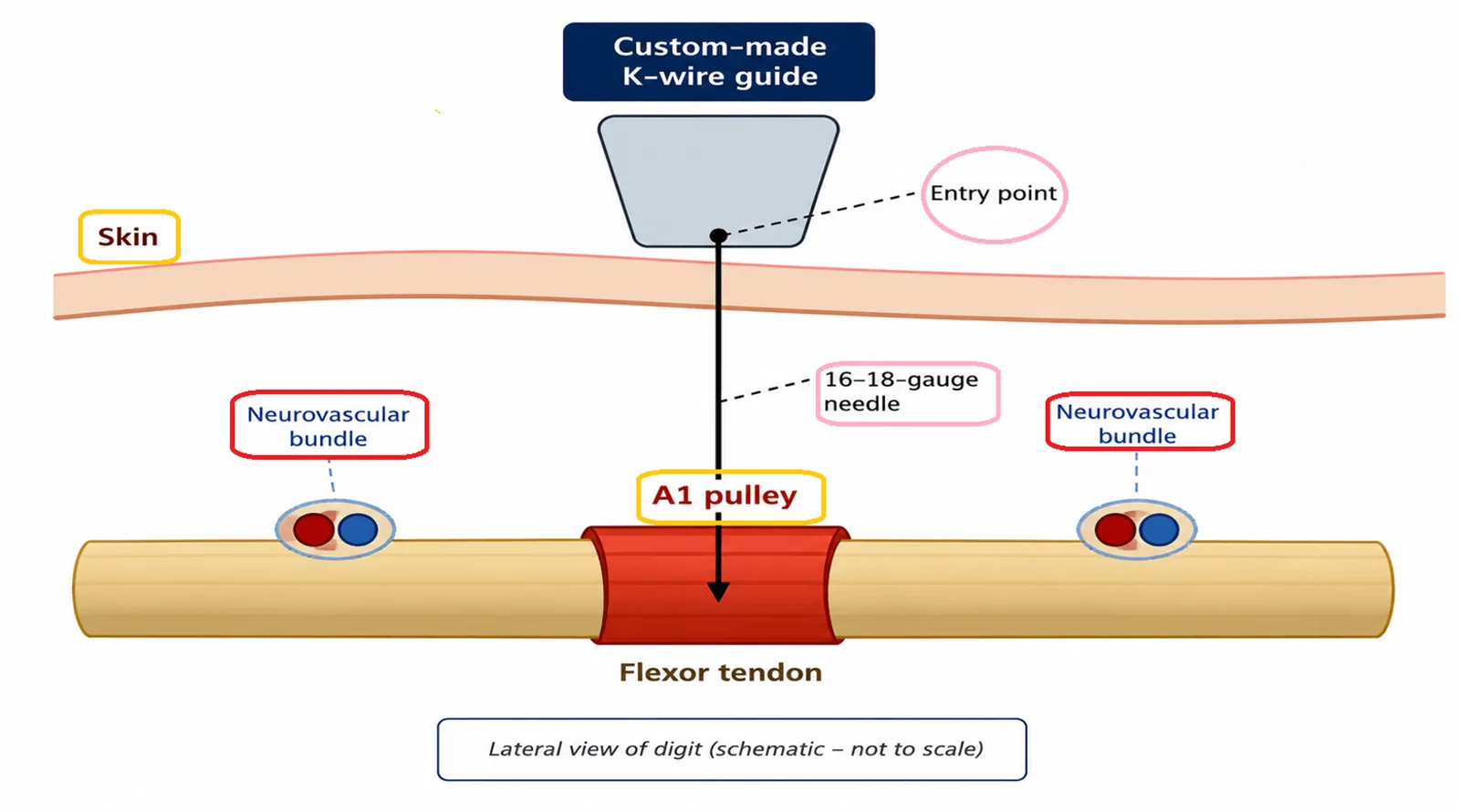
Schematic of guide-assisted needle trajectory Schematic of the guide-assisted needle trajectory relative to the A1 pulley and flexor tendon (lateral view of the digit). The custom-made K-wire guide is positioned against the skin to define a fixed entry point, angle, and depth, constraining the 16-gauge needle to a trajectory at the level of the A1 pulley, kept parallel to the flexor tendon and away from the adjacent neurovascular bundles. This is a schematic representation and not an intraoperative image. The figure was created using Microsoft Word (Microsoft Corp., Redmond, Washington, USA). K-wire: Kirschner wire

Follow-up

Follow-up was conducted through a combination of in-person clinic visits, which included clinical re-examination, and telephone contact for patients unable to attend in person. Telephone-obtained findings were based on patient self-report and were not independently verified by physical examination. The clinic record does not permit a precise, retrospective tally of how many assessments at each follow-up interval were conducted in person versus by telephone; we note this as a limitation, particularly for the longer interval assessments, where telephone follow-up is more likely to have been used.

Outcome measures

Primary outcomes were resolution of triggering, functional recovery (Quinnell grade, active range of motion, VAS pain score), recurrence, and patient satisfaction. Secondary outcomes were complication rate, need for additional or open surgery, and procedure duration.

Patient satisfaction was assessed through a non-standardized verbal clinical evaluation conducted by the treating team at follow-up (in-clinic or by telephone), rather than through a validated satisfaction instrument. This assessment is reported here as a non-validated patient satisfaction assessment and should be interpreted accordingly, particularly given that four participants had a pre-existing relationship with the treating surgeon (see Limitations).

Complications were classified as major (digital nerve injury, tendon injury or rupture, infection, vascular injury) or minor (hematoma, transient numbness, persistent tenderness, skin injury, prolonged pain, stiffness, incomplete release). Minor complications were actively assessed at each follow-up encounter; the only minor finding recorded across the cohort was moderate postoperative pain resolving within five days of the procedure in a subset of patients, the exact number of which was not separately tabulated in the clinical record and is therefore not reported as a precise count. Follow-up assessment specifically and actively confirmed the absence of bleeding, infection, restriction of active range of motion, swelling, and redness at the time points assessed. No major complications were documented.

Because some patients contributed more than one treated digit, digit-level outcomes reported below should not be interpreted as fully independent observations.

Statistical analysis

Data are presented as descriptive statistics: continuous variables as means with standard deviations (and median with range or interquartile range (IQR) where distribution is skewed, as for follow-up duration), and categorical variables as frequencies and percentages. Because of the small sample size and descriptive nature of this retrospective case series, no inferential statistical analyses or formal time-to-event (survival) analysis for recurrence were performed.

Ethical considerations

This study was conducted at Al-Shifa Hospital in Gaza City. No independent institutional review board (IRB) approval was required for this retrospective study. Institutional authorization and approval from the Palestinian Ministry of Health were obtained instead.

Of the 13 patients in this cohort, 4 (30.8%) had a pre-existing professional or familial relationship with the treating surgeon before undergoing the procedure: three were professional colleagues (healthcare workers), and one was an immediate family member (his mother). All were fully informed of the nature of the procedure and its risks and provided consent. All four met the same inclusion criteria and underwent the same standardized technique, data collection, and follow-up described above. We disclose this openly and discuss its methodological implications in the Limitations section, where we treat it as a major rather than a minor limitation of this study's independence.

## Results

A total of 13 patients involving 26 trigger digits were included in this retrospective observational case series. The mean age was 50.5 ± 9.5 years (range 34-65 years). Seven patients (53.8%) were male and six (46.2%) were female (Table 1). Comorbidities (diabetes mellitus, hypertension, thyroid disease, rheumatoid arthritis, asthma) and smoking status were collected as part of the clinical record for all patients; however, individual comorbidity fields were not consistently completed across the full 10-year retrospective record in a manner that allows a reliable tabulated count, and we report this as a data-completeness limitation rather than presenting partial or reconstructed figures (see Limitations). Occupation was recorded for all patients; three patients were healthcare workers who were professional colleagues of the treating surgeon (see Ethical Considerations), and one was an immediate family member. A complete tabulation of occupational categories across the full cohort was not consistently available in a standardized format and is not reported in tabular form for this reason. 

**Table 1:** Baseline demographic and clinical characteristics of patients. Data are presented as mean ± standard deviation (SD) or number (%). VAS (0-10) was used to assess preoperative pain intensity, with higher scores indicating greater pain severity. Comorbidity data (diabetes mellitus, hypertension, thyroid disease, rheumatoid arthritis, asthma, smoking status) and detailed occupational categories were collected as part of the clinical record but were not consistently and completely documented across the full 10-year retrospective period in a format allowing reliable tabulation; this is reported as a limitation rather than presented as partial data. VAS: Visual Analog Scale

Characteristic	Value
Number of patients	13
Number of treated digits	26
Mean age, years (mean ± SD)	50.5 ± 9.5
Male patients, n (%)	7 (53.8)
Female patients, n (%)	6 (46.2)
Healthcare-worker status (colleagues of treating surgeon), n (%)	3 (23.1)
Immediate family member of treating surgeon, n (%)	1 (7.7)
Preoperative VAS pain score (mean ± SD)	6.3 ± 1.0

Twenty-six digits underwent modified percutaneous trigger finger release using the custom-made K-wire guide (Table 2). Of the 26 digits, 17 (65.4%) were located in the right hand and nine (34.6%) in the left hand. By digit, nine (34.6%) involved the middle finger, seven (26.9%) the thumb, five (19.2%) the ring finger, three (11.5%) the index finger, and two (7.7%) the little finger. Preoperatively, 14 digits (53.8%) were classified as Quinnell grade III and 12 (46.2%) as grade IV; no grade I-II digits were included, consistent with the eligibility criteria described in Methods. The mean preoperative VAS pain score was 6.3 ± 1.0.

**Table 2:** Clinical characteristics of treated digits. Anatomical distribution (hand and digit) and preoperative Quinnell grade of the 26 trigger digits treated with modified guide-assisted percutaneous release. Data are presented as number (%). All treated digits were Quinnell grade III or IV preoperatively, consistent with the eligibility criteria described in Methods (refractory to conservative treatment or elective surgical release); no grade I-II digits were included in this cohort.

Variable	n (%)
Right hand	17 (65.4)
Left hand	9 (34.6)
Thumb	7 (26.9)
Index finger	3 (11.5)
Middle finger	9 (34.6)
Ring finger	5 (19.2)
Little finger	2 (7.7)
Quinnell grade III (preoperative)	14 (53.8)
Quinnell grade IV (preoperative)	12 (46.2)

Immediate procedural success, defined as complete disappearance of triggering during active flexion and extension immediately after release, was achieved in all treated digits (26/26, 100%). Procedure duration ranged from approximately 3 to 7 minutes per digit.

At final follow-up, all digits demonstrated full active range of motion, Quinnell grade 0, and a VAS score of 0. No recurrence, repeat procedure, or conversion to open surgery was observed during the available follow-up period (Table 3). Follow-up duration ranged from 3 months to 10 years across the cohort; exact per-digit follow-up dates were not recorded in the retrospective record in a format that permits calculation of a reliable median, interquartile range, or category-by-category breakdown, and this is reported as an explicit limitation rather than presented as an estimated distribution. Because follow-up duration varied substantially and could not be precisely quantified, the absence of recurrence should be interpreted as the absence of observed recurrence during variable surveillance windows, not as evidence that the technique eliminates recurrence risk.

**Table 3:** Surgical and functional outcomes. Outcomes of the 26 treated digits during available follow-up (range: 3 months to 10 years). Visual Analog Scale (0-10). Quinnell grade 0 indicates complete resolution of triggering with full active range of motion. VAS: Visual Analog Scale

Outcome	Result
Immediate procedural success	26/26 (100%)
Full active range of motion at final follow-up	26/26 (100%)
Final Quinnell grade 0	26/26 (100%)
Final VAS = 0	26/26 (100%)
Recurrence during available follow-up	0 (0%)
Repeat procedure	0 (0%)
Conversion to open surgery	0 (0%)
Minor complications (transient postoperative pain, resolved by day 5)	Observed in a subset of patients; exact count not separately tabulated in the retrospective record
Major complications	0 (0%)
Non-validated patient satisfaction ("satisfied" or "very satisfied")	13/13 (100%)

A non-validated clinical satisfaction assessment, conducted verbally by the treating team at follow-up, found that all 13 patients reported satisfaction: 11 (84.6%) "very satisfied" and 2 (15.4%) "satisfied," with none reporting dissatisfaction (Figure 3). Assessment modality (in-person versus telephone) was not recorded in a standardized, tabulable format across the retrospective record and is therefore not reported as a precise count; both modalities were used, as described in Methods.

**Figure 3:**
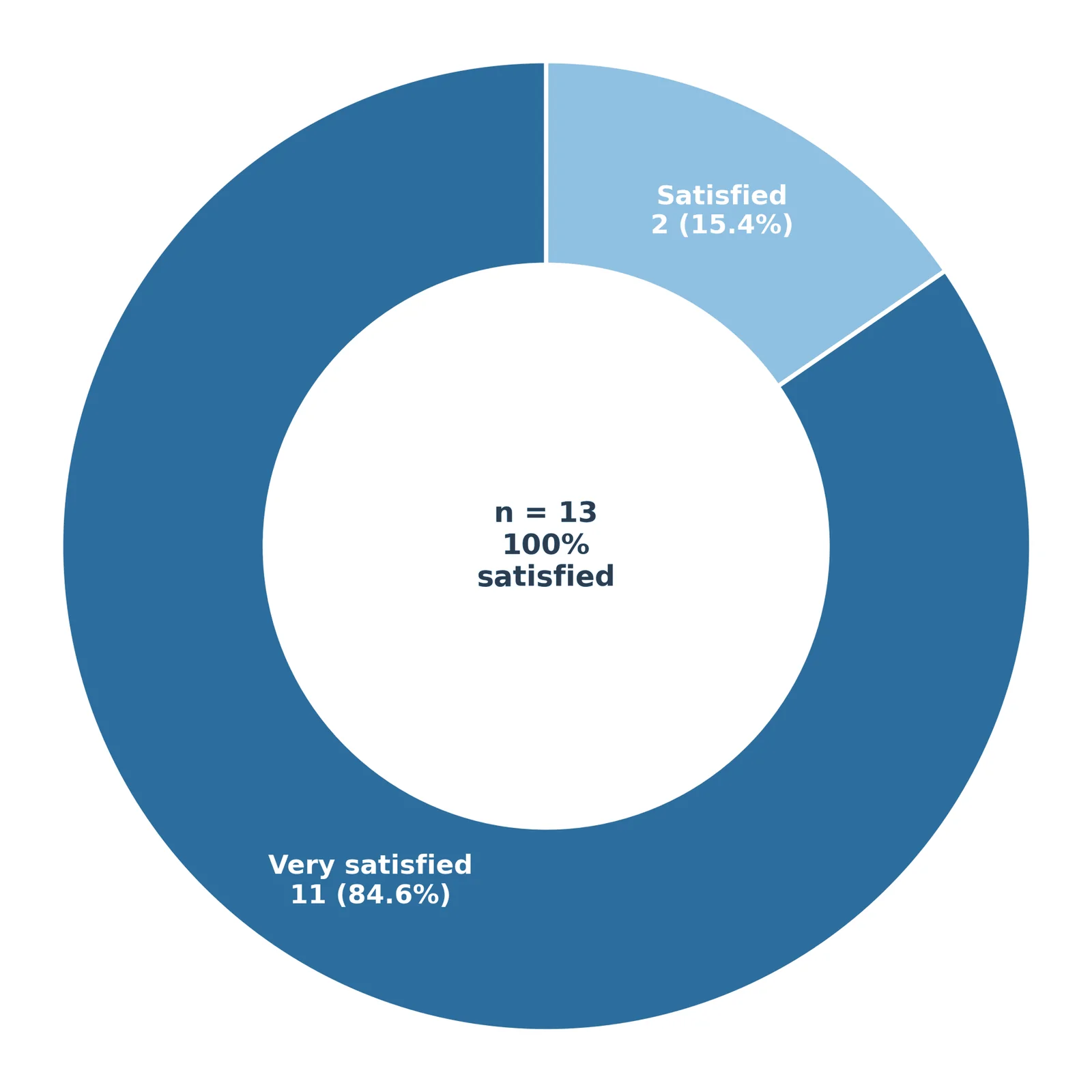
Overall patient satisfaction at final follow-up Donut chart illustrating overall patient satisfaction at final follow-up (n = 13). All patients (100%) reported being satisfied with the outcome of the procedure: 11 patients (84.6%) reported being "very satisfied," and 2 patients (15.4%) reported being "satisfied." No patient reported dissatisfaction.

Minor complications were actively assessed at each follow-up encounter; the only finding recorded across the cohort was moderate postoperative pain resolving within five days of the procedure in a subset of patients, the exact number of which was not separately tabulated in the retrospective record. Follow-up assessment specifically and actively confirmed the absence of bleeding, infection, restriction of active range of motion, swelling, and redness at the time points assessed. No major complications were documented.

Because some patients contributed more than one treated digit, digit-level outcomes (Table 3) should not be interpreted as fully independent observations.

## Discussion

This case series describes the feasibility and observed clinical outcomes of a modified percutaneous trigger finger release using a custom-made K-wire guide. All treated digits achieved immediate procedural success with complete resolution of triggering, and no recurrence or conversion to open surgery was observed during the available follow-up period, which extended up to 10 years for some patients. These observations are consistent with previously published series reporting high success rates for percutaneous release [3,5], although this study cannot establish comparative efficacy, superiority, or durability, given its small size and uncontrolled design. A particular strength of the technique is its simplicity and modest resource requirements: it can be performed in an outpatient setting under local anesthesia using inexpensive, widely available instruments, without reliance on operating-room time, fluoroscopy, or ultrasound equipment. This makes it a technique of interest for resource-limited healthcare systems such as that of the Gaza Strip, where access to surgical facilities and imaging equipment is frequently constrained. The custom K-wire guide was designed to improve consistency of needle trajectory and may potentially reduce procedure-related risk relative to free-hand needle techniques; however, its effect on complication rates cannot be established from this uncontrolled case series, since there was no free-hand comparison group, ultrasound confirmation, or anatomical validation. The follow-up available for this cohort, in some cases extending to a decade, is longer than that reported in many comparable series and is a notable feature of this report. However, because the technique was applied continuously from 2016 to 2026, patients treated later in this window necessarily contributed shorter follow-up, and only a subset of the cohort could have reached the 10-year mark by the close of the study period; we were unable to retrieve exact per-digit follow-up dates from the retrospective record to quantify this distribution precisely. We have therefore avoided framing this as a study that establishes long-term efficacy for the cohort as a whole, and instead present it as a case series with variable, in some cases extended, follow-up, while flagging the inability to precisely quantify that variability as a limitation.

What this study does and does not demonstrate?

We wish to state plainly what these findings can and cannot support. This study demonstrates the feasibility of a low-cost, guide-assisted percutaneous technique and describes favorable observed outcomes in a small, uncontrolled cohort. It does not demonstrate comparative efficacy, superiority over open or free-hand percutaneous release, or a causal safety benefit attributable specifically to the K-wire guide. The 2024 meta-analysis by Casey et al., comprising eight randomized controlled trials and 548 patients, found no significant differences between open and percutaneous techniques in revision rate, complications, or postoperative pain [7]; our data do not challenge or extend that finding, and this study should be interpreted as a feasibility and observational outcome series rather than evidence of superiority over conventional open or percutaneous release. The 2025 randomized controlled trial by Öner et al., comparing ultrasound-guided percutaneous release with open release in 146 patients, found broadly comparable outcomes at 12 months but recorded four revisions in the ultrasound-guided group, related to incomplete release, recurrent tenosynovitis, flexor tendon rupture, and neurovascular injury [8]. This finding is relevant to our rationale rather than to our conclusions: if ultrasound guidance is unavailable, as is frequently the case in Gaza, can a simple mechanical guide provide adequate procedural control? This study was not designed to answer that question directly, since it lacks a free-hand comparison arm, but it motivates a specific, testable hypothesis for future prospective work: a comparison of free-hand percutaneous release versus K-wire-guided percutaneous release, assessing complete release, procedure time, pain, complications, nerve injury, incomplete release, recurrence, and patient satisfaction (Table 4).

**Table 4:** Comparison of the present study with previously published studies on percutaneous trigger finger release Comparison of technique, sample size, follow-up duration, clinical success, and major complications between the present study and selected previously published series on percutaneous trigger finger release [3,4,8]. *Sample size and follow-up duration for comparator studies are reported as stated in the original publications and were not independently verified by the present authors. VAS: Visual Analogue Scale

Study	Technique	Sample	Follow-up	Clinical success	Major complications
Panghate et al. (2020) [5]	Percutaneous release using a 20-gauge hypodermic needle	52 trigger digits*	Short-term (approximately 6 months)*	High success with significant improvement in Quinnell grade and VAS pain score	No major complications reported
Chanthanapodi and Aodsup (2025) [3]	Conventional percutaneous release compared with open surgery	Propensity score-matched cohort*	Long-term	97.6% success; no recurrence or nerve injury reported	No major complications reported
Moungondo et al. (2024) [9]	Retrograde ultrasound-assisted percutaneous release using dedicated sono-instruments	Technical report	Technical description	Demonstrated technical feasibility and emphasized procedural safety	No major complications reported
Present study	Modified guide-assisted percutaneous release using a custom-made 1.0-mm K-wire guide and a 16-18-gauge needle	13 patients (26 trigger digits)	3 months to 10 years	100% immediate release, complete symptom resolution, no recurrence	No major complications, no repeat procedures, and no conversion to open surgery

Previous studies have demonstrated the effectiveness of conventional percutaneous release, while the present study describes a low-cost, custom-made K-wire guide with long-term follow-up of up to 10 years in a resource-limited setting. The custom-made K-wire guide was developed to improve consistency in needle positioning using inexpensive and widely available materials. Although the present study was not designed to compare techniques directly, the favorable outcomes observed support further prospective comparative evaluation.

Strengths

The principal strengths of this study are the extended follow-up available for a subset of patients (up to 10 years, considerably longer than most comparable series), the completeness of follow-up data across all treated digits, and the use of a standardized, guide-assisted technique, which reduces operator-dependent variability compared with free-hand percutaneous methods and may support more consistent reproduction of results, including by surgeons with less experience in percutaneous hand procedures - a hypothesis that remains to be tested prospectively.

Resource considerations

Although a formal cost-effectiveness analysis was outside the scope of this study, the technique requires only a disposable needle, local anesthetic, and a reusable, locally manufactured K-wire guide costing approximately $2 (USD), and does not require an operating theatre, general or regional anesthesia, fluoroscopy, or ultrasound guidance. It does still require sterile preparation, infection-control measures, trained personnel, and sterilization of the reusable guide between uses. We describe the technique as resource-efficient rather than cost-effective, since we did not perform a formal economic analysis. Table 5 summarizes resource requirements relative to open release.

**Table 5:** Resource comparison between the modified percutaneous technique and open trigger finger release Resource requirements are presented for the modified percutaneous technique evaluated in the present study compared with conventional open trigger finger release. The custom-made guide is reusable and was estimated to cost approximately US $2 to manufacture. Procedure time for the modified technique reflects the observed range in the present series; operative time for open release was not measured in this study and is therefore not directly compared.

Resource/requirement	Modified percutaneous technique	Open release
Operating room	No	Usually required
Ultrasound	No	No
Fluoroscopy	No	No
Local anesthesia	Yes	Yes
Disposable needle	Yes	No
Custom-made guide (~US $2; reusable)	Yes	No
Sutures	No	Yes
Operating room staff	Minimal	More extensive
Estimated procedure time	~3-7 min/digit	Not directly measured in this study; generally requires longer operative and perioperative time because of formal surgical setup

Limitations and generalizability

This study has several important limitations. The sample size is small (13 patients, 26 digits), there is no comparison group treated with open surgery or free-hand percutaneous release, and the work was conducted at a single center by a single experienced hand surgeon, all of which limit generalizability and reproducibility by others. As a retrospective case series, it is subject to selection and recall bias, and outcome assessment was not blinded. A substantial and important limitation is that 4 of the 13 patients (30.8%) had a pre-existing professional or familial relationship with the treating surgeon before undergoing the procedure (three professional colleagues and one immediate family member). We consider this a major, rather than minor, limitation, since it introduces the potential for selection bias, relationship bias, reporting bias, satisfaction bias, reluctance to report complications, preferential follow-up, and perceived pressure to report favorable outcomes. All four patients had excellent outcomes and reported satisfaction, which makes this potential source of bias particularly relevant to the interpretation of our results. Patient satisfaction was assessed using a non-validated, verbal, investigator-administered clinical question rather than a validated instrument, and this limitation is compounded by the relationship bias described above. Similarly, this study lacks a validated, standardized hand-function questionnaire (e.g., QuickDASH, DASH, PROMIS, or the Michigan Hand Questionnaire); outcomes were instead captured using Quinnell grading, VAS pain scores, and active range of motion, which do not fully capture patient-reported functional status. Full active range of motion does not necessarily establish complete functional recovery, since patients could still have grip weakness, residual tenderness, scar sensitivity, or activity limitations that were not systematically assessed. The absence of recurrence in this small cohort during the available follow-up should not be interpreted as evidence that the technique eliminates recurrence risk; with only 26 treated digits, the confidence interval around a zero-event estimate is wide, and recurrence surveillance duration varied substantially across the cohort given follow-up ranging from 3 months to 10 years. No formal time-to-event analysis was performed given the small sample size and the inability to retrieve exact per-digit follow-up dates from the retrospective record. A chronological (early-versus-late case) comparison to evaluate a possible learning-curve effect was not feasible to extract reliably from the retrospective record; we recommend this as part of future prospective comparative work. Analgesic requirements were not systematically recorded in the clinical charts for this retrospective series and could not be reliably reconstructed, and are therefore not reported. Nonetheless, the consistency of observed outcomes across all 26 treated digits, together with the follow-up period available for a subset of patients, provides encouraging, hypothesis-generating evidence regarding the feasibility of this technique. Future prospective, comparative studies with larger cohorts, a free-hand percutaneous release control group, standardized patient-reported outcome instruments, blinded outcome assessment, and formal cost analysis are recommended to validate these observations and to directly compare this modified technique against open release and free-hand percutaneous approaches.

## Conclusions

Modified guide-assisted percutaneous trigger finger release using a custom-made 1.0-mm K-wire guide appears feasible and was associated with favorable observed outcomes in this small, retrospective, uncontrolled case series, including complete symptom resolution, restoration of full active range of motion, and high patient-reported satisfaction on a non-validated assessment, with no recurrence or major complications observed during the available follow-up (up to 10 years for a subset of patients). This should be interpreted as a feasibility and outcome case series rather than evidence of superiority over open or free-hand percutaneous release. Larger, prospective, controlled studies - ideally comparing free-hand and K-wire-guided percutaneous release, with validated functional outcome measures and blinded assessment - are warranted to establish the safety, effectiveness, and reproducibility of this low-resource technique, particularly for resource-constrained healthcare settings.
